# Conventional and modified hydrodistillation method for the extraction of glucosinolate hydrolytic products: a comparative account

**DOI:** 10.1186/s40064-016-2021-z

**Published:** 2016-04-18

**Authors:** Rohit Arora, Bikram Singh, Adarsh Pal Vig, Saroj Arora

**Affiliations:** Department of Botanical and Environmental Sciences, Guru Nanak Dev University, Amritsar, Punjab 143005 India; Natural Plant Products Division, CSIR-Institute of Himalayan Bioresource and Technology, Palampur, Himachal Pradesh 176061 India

**Keywords:** Hydrodistillation, Magnetic stirrer with hot plate, Glucosinolates, Isothiocyanates, Erucin, GC–MS

## Abstract

*Eruca sativa* is extensively used as raw and its oil is also used for cooking due to its exceptional flavour. The volatile nature of the hydrolytic products of glucosinolates makes the extraction difficult. The hydrodistillation method used previously yield very less amount of the extract as well as the absence of stirring in the round bottom flask causes burning of both the crushed seeds and the flask. To overcome these drawbacks, a method has been developed using magnetic stirrer and hot plate. The yield and composition of hydrolytic products in the extract with the modified method was increased along with an increase in the amount of major hydrolytic products as seen by GC–MS. This method thus has immense potential in pharmaceutical industries, due to the ease of extraction and isolation.

## Background

*Eruca sativa*- an annual herb of Mediterranean origin is rich in glucosinolates and is commonly known as arugula, rocket salad, rugula or taramira (Khoobchandani et al. 2010; Arora et al. 2014a, b). Its aerial portion has been used extensively in most parts of the world as salads. The oil obtained from their seeds is used in pickles and for cooking purpose. A number of reports are available that signify the biological importance of the glucosinolate hydrolytic products (Khoobchandani et al. 2010; Vig et al. 2009). These varied biological activities form the basis for developing the appropriate methods to achieve their maximum yield as well as number. A thorough literature survey reveals that although cold as well as hot extraction methods were used to obtain the valuable glucosinolate hydrolytic products (GHPs) from this plant, but it was seen that cold extraction methods were time consuming, gave a lower yield and that too with other unwanted compounds (other than glucosinolate hydrolytic products). On the other hand, the volatile nature of the compounds made hydrodistillation an important choice for the extraction. It gave a much higher yield in lesser time with lesser number of undesirable compounds. The only problem with this method was that it was not effective for the extraction of compounds from seeds. The reason being that the seeds when crushed absorb a lot of water and become heavier, thus crushed seeds settle down at the bottom of the round bottom flask. This causes the burning of the seed powder as well as the flask. The burning of the seed powder affects the yield as well as number of GHPs obtained.

In order to get rid of the above problems, a number of methods were used viz. microwave assisted and supercritical fluid based hydrodistillation (Arranz et al. 2015; Patra et al. 2015). Although the methods were effective but they involve the use of complex and highly expensive instruments. Keeping these facts in mind, a hydrodistillation method with magnetic stirrer and hot plate was developed that involved cheaper and simple instrument and gave much higher yield in lesser time. Further, the amount of the major components of the extract was very high making it easier to isolate them.

## Methods

### Conventional hydrodistillation

**Fig. 1:**
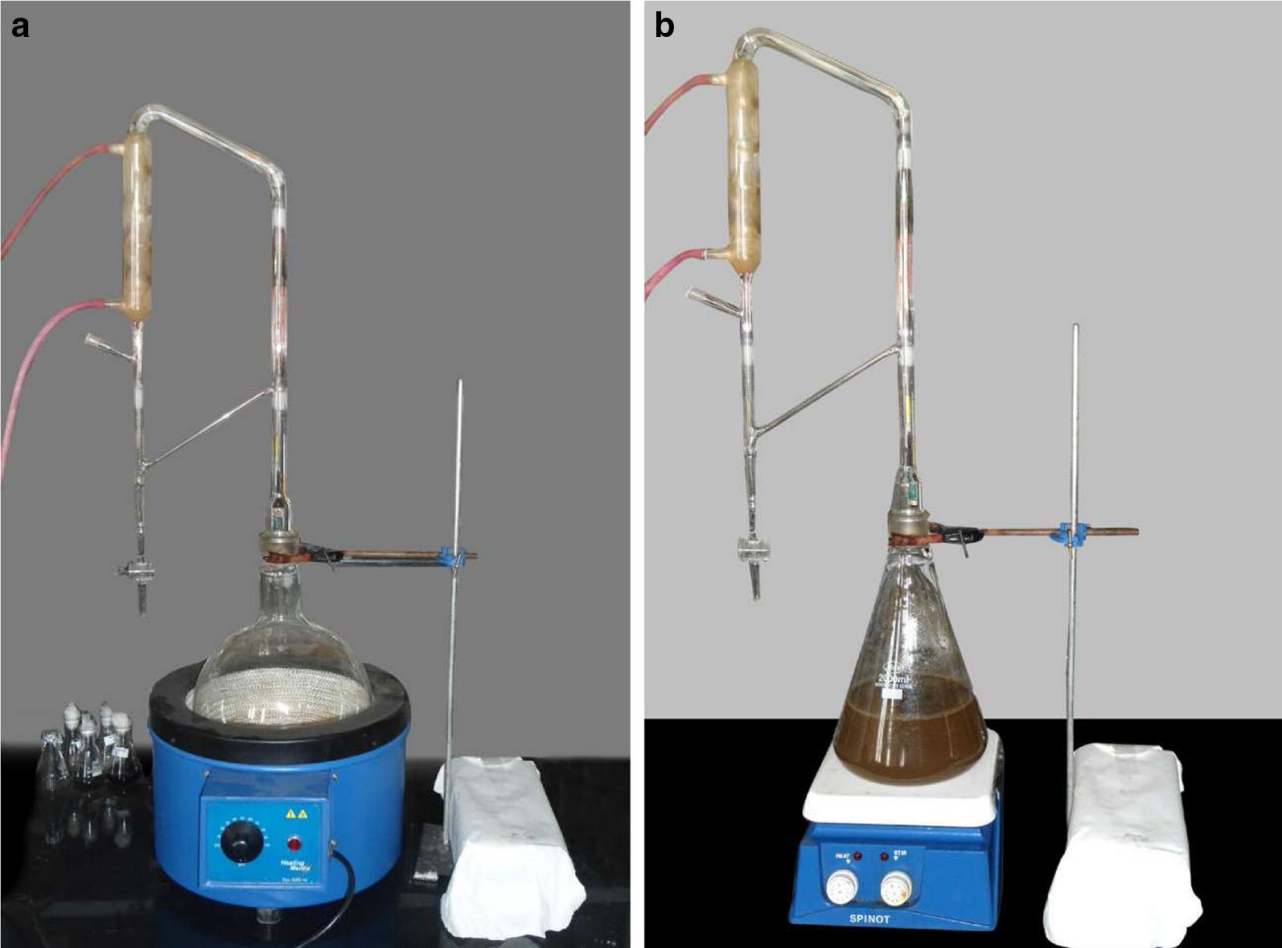
The different hydrodistillations methods where **a** conventional method and **b** modified method

The conventional hydrodistillation was done following the method given by Blazevic and Mastelic (Blazevic and Mastelic 2009). In this, 50 g seeds of *Eruca sativa* (Mill.) Thell. var. RTM-1212 (procured from Sri Karan Narendra College of Agriculture, Jobner, Rajasthan, India), were crushed and added to 1000 ml distilled water (DW) in a round bottom flask. The flask was then kept in a heating mantle and the Clevenger apparatus was attached (Fig. 1a). The mixture was allowed to boil at 100 °C and then the temperature was reduced to 60 °C and kept for 3 h. The oil and water obtained was collected in a separation flask and the GHPs were obtained by fractionation using AR grade dichloromethane (DCM). The solvent was passed through anhydrous sodium sulphate and evaporated under vacuum at 30 °C, using rotary evaporator (Buchi R210). It was redissolved in GC grade DCM and passed through 0.22 μm filter prior to GC–MS injection.

### Modified hydrodistillation

The modified hydrodistillation method involved a magnetic stirrer with hotplate (Spinot, Tarsons Products Pvt. Ltd., New Delhi, India). Here, 50 g crushed seeds of *Eruca sativa* (Mill.) Thell. var. RTM-1212 were added to 1000 ml DW in a flat bottom flask containing a magnetic stir bar. The flask was attached to a clamp stand and the Clevenger apparatus was attached to it (Fig. 1b). The mixture was allowed to boil at 100 °C with rotation set at 400 rpm. Later, the temperature was reduced to 60 °C and rotation was kept at 300 rpm for 2 h. The oil and water mixture was collected and processed as described in “Conventional hydrodistillation” section.

### Yield

The yield of the final extract obtained by the two hydrodistillation methods was calculated using the weight of the seeds and the extract obtained from it.

### Gas chromatography–mass spectrometry

The GC–MS analysis of the extracts was carried on Shimadzu (QP2010 series) gas chromatograph–mass spectrometer (Tokyo, Japan) equipped with an AOC-20i auto-sampler coupled to a DB-5 MS capillary column (30 m × 0.25 mm i.d., 0.25 μm). The injection temperature was 40 °C and the volume of the sample was 2 μl. An initial temperature of 40 °C was maintained for 4 min, followed by a gradient increase to 230 °C at the rate of 4 °C/min and the temperature was held at 230 °C for 15 min. Inlet pressure was kept at 97.1 kPa with helium as a carrier gas released at a flow rate of 1.1 ml/min in split mode (1:50). The MS was set with an interface temperature of 250 °C at EI mode of MS and the detector voltage of 0.9 kV. The mass range was 40–800 u with the scan speed of 1666 u/s at an interval of 0.50 s (2 Hz).

## Results and discussion

**Fig. 2:**
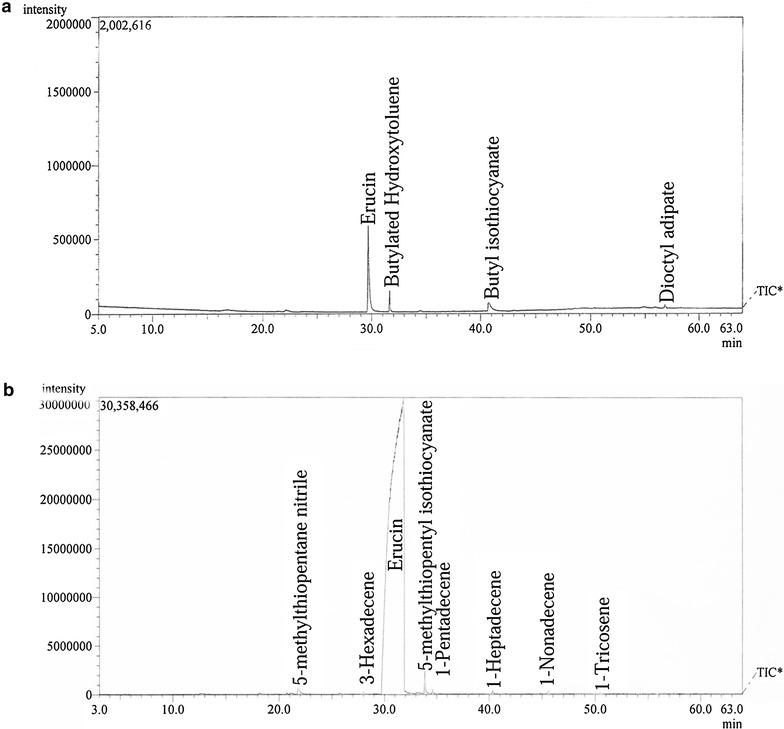
The GC–MS analysis of the extract, where **a** conventional method and **b** modified method

The conventional hydrodistillation method gave a yield of 0.825 % with the composition of 4-methylthiobutyl isothiocyanate (erucin) (76.31 %) and butyl isothiocyanate (11.87 %) (Fig. 2a). Erucin was isolated from the extract, since it is the major GHP of *E. sativa* (Arora et al. 2014b). The yield obtained was comparatively less with only two hydrolytic products in it. Some compounds other than glucosinolate hydrolytic products were also obtained during the extraction as detected in the extract, which were butylated hydroxytoluene (9.22 %) and dioctyl adipate (2.60 %) (Fig. 2a). The composition of hydrolytic products is only two and in addition the other undesirable compounds (total 11.82 % of extract) present may act in an antagonistic or synergistic way to affect the activity of the extract. The Butylated hydroxytoluene is often used as a food additive and has antioxidative properties (Aourahoun et al. 2014). On the other hand, dioctyl adipate is a plasticizer and is toxic at certain levels (Milkov et al. 1973). These together may hinder the actual activity of the extract and may also cause slight toxicity.

The refined hydrodistillation was designed to solve the major problem of burning of the seeds by stirring the seed mixture. In addition to the mixing, a homogenous supply of heat to the entire mixture was ensured. A yield of 0.910 % was achieved by the modified hydrodistillation method, which was slightly higher than the conventional method. The time required in obtaining the final extract was also reduced. In this case as well, erucin was found to be the major hydrolytic product with the concentration of 98.77 % of the extract. There were two more hydrolytic products present viz. 5-methylthiopentane nitrile (0.29 %) and 5-methylthiopentyl isothiocyanate (0.57 %). A few compounds other than the glucosinolate hydrolytic products were also present in the extract viz. 3-hexadecene (0.06 %), 1-pentadecene (0.10 %), 1-heptadecene (0.08 %), 1-nonadecene (0.09 %) and 1-tricosene (0.04 %). The total amount of these undesirable compounds was only 0.37 % of the total extract, which is quite negligible in comparison to the major hydrolytic product. The amount of major hydrolytic product i.e. erucin in the extract, makes it quite suitable for the isolation purpose and thus the current hydrodistillation method can further be employed for obtaining purified compounds.

## Conclusion

The modified hydrodistillation method involving magnetic stirrer with hot plate provided better yield and composition of GHPs in lesser time in contrast to the conventional method. Moreover, the amount of major hydrolytic product i.e. erucin was significantly higher than the conventional method. This in turn simplifies the isolation of an important and biologically active compound, thus making it suitable for laboratory purposes.
